# Usability Testing an mHealth Program with Tailored Motivational Messages for Early Adolescents

**DOI:** 10.3390/nu15030574

**Published:** 2023-01-21

**Authors:** Carolyn A. Lin, Kayla L. Vosburgh, Deya Roy, Valerie B. Duffy

**Affiliations:** 1 Department of Communication, University of CT, Storrs, CT 06269, USA; 2 Department of Allied Health Sciences, University of CT, Storrs, CT 06269, USA; 3 Department of Communication, California State University San Marcos, 333 S. Twin Oaks Valley Rd, San Marcos, CA 92096, USA

**Keywords:** health communication, healthy eating, active living, tailored messages, adolescent, health interventions, mHealth application, mobile app usability, pediatric obesity prevention

## Abstract

Obesity among children is a rising concern throughout the world. In the U.S., rates of childhood obesity are the highest among children from diverse and economically disadvantaged households. Obesity in adolescence increases the risk of negative physical and psychological health consequences. Mobile-app-based health interventions have been found to be an effective tool to encourage children to adopt a healthier living style. A novel mobile app prototype was developed for early adolescents to communicate with and engage them interactively about healthy eating and active living. To test the app’s usability, students from a U.S. middle school, with a majority of children from low-income families, were recruited to use the app and report their feedback. The usability testing results confirmed that the app was equally well received by participants of different genders, normal weight versus overweight/obesity, and amounts of screen time. Study participants also provided overwhelming positive feedback for the embedded and tailored motivational messages that encourage healthy eating and active living. The conceptualization of the app prototype was guided by the self-determination theory, social cognitive theory, and priming theory, in addition to incorporating evidence-based obesity prevention principles. This prototype, hence, provides a valid platform for building theory-based behavioral interventions.

## 1. Introduction

The prevalence of obesity among children and adolescents ranged from 20% to 22% during 2017–2020 in the United States [1]. Obesity in children can cause health concerns, such as type 2 diabetes, asthma, nonalcoholic fatty liver disease, musculoskeletal disorders, mood disturbances, polycystic ovarian syndrome, and metabolic syndrome [2]. Childhood obesity may also result in social isolation and social discrimination, which can lead to undesirable psychological outcomes, such as negative body image, lower self-esteem, and depression [3]. Research has also shown that adolescents with overweight or obesity often grow up to become obese or overweight adults [4].

Certain groups of children are more likely to experience obesity due to socioeconomic challenges. For example, middle school students from economically disadvantaged households were more likely to live with overweight or obesity, relative to those from families with more financial security [5]. Likewise, prior work also found that food insecurity and obesity were correlated among children from low-income households [6]. To reduce the risk of obesity in adolescence, schools are regarded as an important setting for promoting healthy foods [7], physical activity [8], family meals and avoiding weight stigma and negative dieting behaviors [9].

In particular, working with schools is a viable option to integrate health promotion and intervention programs that leverages technology into the health education curriculum. For instance, low- (N = 310) to middle-income (N = 195) middle school students responded favorably to tailored motivating or reinforcing health messages (for behavioral change)—that were interactively triggered by and individually delivered based on their online survey responses—which assessed their likes/dislikes of foods/beverages and physical/sedentary activities, sleep indicators, and perceived food insecurity [10]. However, this type of intervention may not be seen as personal enough to fully engage study participants at an individual level.

mHealth technology that utilizes mobile phones (commonly considered a personal technology device) can help extend the reach of school-based interventions to involve adolescents from disadvantaged families [11], allowing the use of tailored messages to engage the families [12]. As 95% of U.S. teens have access to or own a smartphone and 93% of teens from households earning less than USD 30,000 have reported the same [13], mHealth tools, such as a mobile app, could be an alternative venue—for implementing adolescent obesity prevention strategies—to complement traditional school-based programs.

While there are thousands of mHealth apps on various platforms, scholars have been debating the effectiveness of these apps on users. mHealth apps have the potential to deliver low-cost or no-cost interventions to users on demand [14]. Studies have lent support to the idea that mHealth apps offer compelling benefits ranging from improvement in physical activity [15] to mental health [16]. Considering the availability of smartphones in adolescents, health interventions delivered through mHealth apps have a better reach within this population than do paper-based or face-to-face programs [17].

Against this backdrop, the current study involved usability testing of a novel mobile app prototype developed to communicate with and engage early adolescents (ages 10–14) interactively about healthy eating and active living. These adolescents were students attending a U.S. middle school with the majority coming from low-income households and thus having greater risk of overweight and obesity [5,18]. A recent study also suggested that as food insecurity and childhood obesity exist disproportionately in low-income children, pediatric obesity in this demographic has only been magnified during the recent COVID-19 pandemic [6].

Specifically, the objectives of this study include: (1) testing the perceived usefulness, learnability, ease of use, and user satisfaction associated with the app, and (2) pilot-testing tailored healthy eating/active living messages in response to users’ online-reported eating and exercising habits. It is anticipated that the usability testing results of the current study will help refine the app prototype to produce a useful tool for the children and their families to motivate healthier lifestyles.

## 2. Literature Review

During early adolescence, children experience physical changes and feel self-conscious of their physical growth and body image; they also become interested in learning life skills [19]. As such, early adolescents are an ideal group to receive information that will benefit their desire for acquiring life skills (e.g., self-monitoring, goal setting, and healthy behavior skills) and facilitating their physical and emotional growth. If we assume that most early adolescents can maintain healthy living habits through late adolescence, then they would likely avoid becoming overweight or obese when they transition into adulthood. Moreover, early adolescents are old enough to grasp the concept of beneficial life skills (e.g., healthy living habit) and young (and curious) enough to not rebel against learning such skills relative to older adolescents. For these reasons, the mHealth app prototype examined in the current study was developed to target those early adolescents, ranging in age from 10 to 14 years old.

### 2.1. mHealth Technology and Adolescent Obesity Prevention

As smartphone use is rather common among adults and children, mHealth interventions have a large potential for positive outcomes. mHealth is defined as “the use of mobile communications for health information and services” where the primary goal is to “improve health outcomes” [20]. Considering the easy-to-access and prevalent nature of mobile phones, a mobile app could be an ideal tool that enables app users and researchers to assess or monitor users’ health or lifestyle [21]. For example, the ecological momentary assessment (EMA) methodology [22] enables researchers to analyze lifestyle or activities in real time and in real-world settings [23].

However, the relevant limited literature suggests that commercially available mHealth systems may adhere neither to recommendations nor to techniques rooted in best practices. For example, a study of 57 apps reported that, while 61.4% of commercially available apps did not use any of the 2007 Expert Committee for Pediatric Obesity Prevention (ECPOP) recommendations, the highest-rated app only employed 6 of the 15 recommended targets and strategies [24]. Similar findings were also concluded by another analysis of 62 apps for pediatric obesity prevention, which found low adherence to ECPOP recommendations and only weak, nonspecific promotion of behavioral targets and very few intervention strategies [25].

To enhance the effectiveness of mobile apps utilized for obesity prevention in children, the app design should align with expert recommendations and be grounded in behavioral change theories (BCTs). BCTs have been widely adopted to provide the framework for designing and implementing various types of health intervention programs [26]. For instance, the self-determination theory (SDT) posits that individuals have three basic psychological needs: (1) competence or the need to effectively master an outcome, (2) autonomy or the need to control one’s own future, and (3) relatedness or the desire to connect with others. Obesity interventions reaching children and adolescents have often paired SDT with another theory, such as the social cognitive theory (SCT), which emphasizes the importance of developing one’s self-efficacy to set and accomplish goals [27,28].

Extant research, though preliminary, suggests that relevant apps developed for adolescents have not regularly adopted BCTs in their design. Based on a review of 383 child/adolescent physical activity and dietary apps, only a few incorporated the BCTs that were deemed effective to result in health behavior changes [29]. As few apps targeting obesity prevention behaviors in adolescences incorporate BCTs, it is not surprising that even though many adolescents downloaded health-related mobile apps, only 7% report a subsequent health behavior change [30].

### 2.2. mHealth Technology and Health Program Design

When BCTs are applied to guide the development of a health promotion program, they can better identify which factors influence an individual’s ability and decision to change. The most effective BCTs involve prompts (extra hints or reminders for behavior change) and cues (something in the environment that naturally reminds the person to do the behavior change) [31]. The few apps that included BCTs adopted the transtheoretical model and SDT as a theoretical guide [32], incorporating such concepts as self-monitoring and performance feedback to increase participant awareness and motivate the participants to contemplate and then subsequently engage in behavior change.

For instance, *Creature-101* is a web-based game that promotes healthy eating and physical activity in young adolescents using the SDT and SCT as a framework for the game [28]. Behavioral change techniques included goal setting, motivational messaging, outcome/feedback reinforcement, cues/triggers, and rewards. Participants reported significant decreases in sugar-sweetened beverages and packaged snacks, but no differences in intakes of fruits, vegetables, and water, or the level of physical/sedentary activities. In another example, an SDT- and SCT-guided smartphone app, *ATLAS* (Active Teen Leaders Avoiding Screen-time), was tested among low-income adolescent boys to help them set personal health goals and monitor/track behaviors in a school-based program.

To reach low-income adolescent girls, a standalone app provided BCT features, including cues to action, self-monitoring, feedback, and reinforcements; the study generated moderate effects on increased consumption of fruits and vegetables and decreased consumption of sugar-sweetened beverages [33]. Furthermore, a systematic literature review (N = 41) of mobile apps, games, and text messaging programs for preventing and treating pediatric obesity found no significant improvements in adiposity measures [34]. Results also indicated that these mHealth tools successfully promoted: (1) physical activity, when they included social networking, self-monitoring, and feedback features; (2) increased fruit and vegetable consumption; and (3) behavior change via self-monitoring and goal setting.

Empirical studies have also shown that health priming could reduce unhealthy food consumption behavior. The priming theory is a framework useful in explaining how exposure to health promotion messages may activate cognitive response to influence further information processing and acceptance [35,36]. An assumption of the priming theory is that the individual or target of prevention does not need to be conscious or aware of the influence of the primes (or persuasive messages) when the prime aligns with the individual’s behavioral goal [37,38,39]. For instance, exposure to the cover of a diet and health magazine among those who are dieting led to a decrease in consumption of less healthy foods [40]. Similarly, with exposure to diet cues in a television commercial, dieters chose to reduce consumption of snack food [41]. Likewise, dieters who received primes with their weight control goal also showed less attention to hedonic food cues, compared with dieters in the control group [42]. Similarly, after paying initial attention to the prime (a flyer) received at the beginning of the experiment, overweight and obese participants’ snack purchases in grocery stores were reduced by 75%, when compared with the control participants [43]. A meta-analysis further supported these findings by revealing that weight control priming cues effectively reduced food intake among individuals with weight control goals [44].

By implication, if mobile app users are primed with motivational messages that expose them to choosing a healthier lifestyle during a related task (e.g., reporting their eating and exercising behaviors), then these health primes may direct their attention to making healthier choices. To date, research has yet to apply the priming theory as a framework to help guide the development of mobile health apps.

### 2.3. Current Mobile App and Research Questions

Mobile health apps developed for young users typically aim for adolescents aged 12–19 as the intervention target [45]. Our app prototype was developed to target early adolescents (aged 10–14) to encourage healthy behaviors. The focus on early adolescence allowed for a developmentally appropriate approach, as children in this age group are just starting to become curious and interested in learning about life skills. By focusing on this developmental stage, it also permitted the implementation of age-appropriate motivational messaging as a technique to build self-efficacy related to the adoption of a healthy lifestyle.

The final “release version” of the mobile app (whose prototype usability testing is being reported here) will serve as a free technology tool for aiding teachers in health education classes—and for motivating parents and their children—to learn and practice healthy eating/active living. The published app will also be a free digital tool that can extend the service for the educational arm of programs such as the Supplemental Nutrition Assistance Program (SNAP-Ed) to reach children and families of economic disadvantage. Additionally, this free app will also be promoted to parents and children in the waiting rooms of pediatric clinical settings for on-site and postvisit adoption consideration.

The different content features of this mobile app prototype were designed by fusing the thesis of BCTs described, including the SDT [46], self-efficacy [47] and priming theory [35,36]. Specifically, by interacting with the app features, participants can learn about and practice healthy living behavior—by demonstrating self-monitoring behavior (i.e., competence), exercising control over their behavioral outcomes (i.e., autonomy), and cultivating social acceptance (if part of a unique mHealth community at school) (i.e., relatedness)—to help build their self-efficacy. In addition, health promotion messages are also strategically embedded in the app to prime adolescents to consider adopting healthy eating and exercising habits through their interaction with the relevant app features (Appendix A). The textual and visual content as well as the interaction flow design was tailored to appeal to early adolescents.

As the study aimed to understand how 10- to 14-year-old app users may interact with the information architecture design and age-appropriate content features, the following research questions were proposed to examine how these pre- and early adolescents may evaluate their user experience. Specifically, a set of research questions was proposed below to query four app usability factors, conceptualized based on two widely adopted instruments: USE Questionnaire [48] and Post-Study System Usability Questionnaire (PSSUQ) [49]. These factors included perceived ease of use, usefulness, learnability, and user satisfaction. In addition, two research questions exploring the priming effect of the health messages and whether usability factors were posed to help explain the perceived effectiveness of those messages.

RQ1a–d: What is the perceived (a) ease of use, (b) usefulness, (c) learnability, and (d) user satisfaction experienced via user interface with the app?

RQ2: What is the perceived effectiveness level of the health promotion messages?

RQ3a–d: Is the perceived level of (a) ease of use, (b) usefulness, (c) learnability, and (d) user satisfaction positively related to perceived health message effectiveness?

## 3. Materials and Methods

Our sample was derived from a Title 1 middle school in the state of Connecticut in the U.S., through our partnerships in delivering nutrition education to income-disadvantaged communities. Title 1 schools are those that have additional federal funding in order to support low-income students [50]. The Title I school where our study data were collected had approximately 69% of the students receiving federally subsidized meals. The protocol was reviewed and approved by the university’s internal review board (IRB) prior to participant recruitment and study implementation.

### 3.1. Study Procedure

Two graduate assistants (both registered dietitians), in collaboration with the health education teacher at the middle school, introduced the mobile app to students in health classes via a 10 min presentation. The presentation explained the purpose of the study and provided a visual demonstration of where and how to download the app. All students in the said class were asked to bring home to their parents/caregivers an information sheet (which included the validated information sheet with the IRB’s stamp used to consent each participant) and enrollment flier (which describes the app with instructions on how to download and log on to the app). Forty-nine students logged in at school with two Android tablets provided by the research team, in order to standardize access to technical features. These tablets were used because school policy disallowed students to use a mobile phone on campus.

After downloading the app, participants were invited to review an information sheet to express their agreement to participate in the study (by checking a box on the sheet) before logging in the study. After successful log-in, the participants were asked to create a nickname ending in the 3-digit number on the randomly distributed enrollment flyer in class. Once the participants had created their own nickname and entered self-reported information on age, gender, height, and weight, they then began to interface with all the features built into the app.

Two weeks after the usability study was concluded, the graduate assistants returned to the health classes to distribute nutrition education reinforcement gifts. Each student was also given a wristband, which had healthy messages regarding nutrition and physical activity embossed on it. These reinforcement gifts were offered to promote a healthy lifestyle to all students, regardless of whether they participated in the usability study. In addition to providing reinforcement gifts, the graduate assistants also offered a nutrition lesson on healthy snacking.

### 3.2. Sample Description

Twenty-seven of the 49 study participants successfully completed the usability study. This sample size is more than adequate to detect potential interface glitches and other architectural problems for a mobile app usability study. The sample size of past usability studies conducted for testing mHealth apps varies from 10 [51] to 23 [52]. Empirical evidence suggests that 5 participants for a usability study is the tried-and-true standard [53]. However, when the sample size becomes larger, the mean frequency for a usability test to identify problems also increases (N = 20, 98.4%, and N = 30, 99%) [54].

On average, it took the participants less than 6 min to create a username, log on, interface with all app features, and respond to the embedded usability measurement items. Based on reporting within the app, the majority of the participants were female (58.3%), 12–13 years old (M = 12.7, SD = 0.73). Based on self-reported height, weight, and age, 26.8% (body mass index > 85th percentile) were estimated as overweight or obese [3]. However, these results should be interpreted with caution, as research shows that children significantly underreport height and weight, although older children tend to report more accurate numbers [55].

### 3.3. App Feature Description

This app contains five feature-interaction and two measurement categories. The first category covers food/beverage consumption and enables the reporting of three meals (i.e., breakfast, lunch, and dinner), beverages (e.g., milk, juices, water, sports drinks, and soda drinks), desserts (e.g., cakes, pies, and ice cream), and snacks (e.g., candy, cookies, popcorn, and fruits) as well as the location of the meals (home, school, and on the go). The second category profiles physical activity and prompts the reporting of the level of bodily movement throughout the day, including different exercises (e.g., gym class), sports (e.g., baseball, basketball, soccer, running tracks, and swimming), and physical movements (e.g., playing outside, walking, and riding a bike to school).

The third category describes screen time use and solicits the reporting of time spent with digital technology devices on a daily basis (e.g., television, video games, computer, and mobile phone) and parental rules for technology use. The fourth category addresses sleep pattern and allows the reporting of bedtime, wake time, sleep duration, and sleep problems. The fifth category asks users to report their current mood (i.e., how they are feeling), favorite foods, beverages, and activities, as well as their perceived vs. desired body image using the child’s “body figure perception” scale [56]. Lastly, the sixth category contains 3 health message effectiveness measures and 12 usability measures (see operational definitions below).

Tailored health promotion messages were programmed to make a pop-up appearance to match user responses to the different feature categories described above (see Appendix A). The purpose of these messages was to motivate self-driven learning and engagement in healthy behaviors. These messages also contain brief, memorable, educational, fun, and engaging child-friendly images (e.g., fruits, foods, and emojis). Consistent with making the app interface friendly and enjoyable, nearly all features and measurement items were engineered to involve a simple click of an image interface (e.g., breakfast cereal, bananas, soccer ball, and bicycle) to allow users to select or enter a response on each screen.

### 3.4. Measures

Usability assessment. Users reported their evaluation of the app’s usability using 12 items, measured on a 7-category facial hedonic scale (7 = biggest smile). These items assess psychometric responses to human–computer interface—USE Questionnaire [48] and PSSUQ [49]. The 12 items reflected 5 conceptual dimensions in the 5 research questions. The “ease of use” dimension contained the items: “This app was easy to use”, “I could answer the questions quickly”, and “I could use this app without help”. The “learnability” dimension included the items: “I could fix my mistakes easily and quickly”, “It’s easy to learn how to use the app”, and “It’s easy to understand the pictures”. The “usefulness” dimension described the items: “The questions asked me about what I do daily”, “This app made me think about what I eat and what I do”, and “There were pictures of what I ate and did”. The “satisfaction” dimension had the items: “I liked using this app”, “I would use this app again”, and “I would tell friends to use this app”.

Health message assessment. Upon completion of interacting with the app features, the users were asked to evaluate the effectiveness associated with the health promotion messages by responding to three statements: (1) I learned new information about food and nutrition from this app, (2) The messages I received were helpful when I made food choices; and (3) I liked getting nutrition messages related to my day. Response categories to these questions were measured using the 7-point facial hedonic scale.

### 3.5. Statistical Analysis

To analyze the study data and test the research questions, the following statistical procedures were adopted. First, an exploratory factor analysis was performed to validate the conceptual dimensions of the usability and health message assessment measures, with principal component extraction and varimax rotation. Second, weight status was computed to identify the percentage of participants within the definition of weight status [3] as healthy weight (>5th to <85th percentile) and overweight/obese (≥85th percentile). Third, descriptive statistics were computed to generate frequencies, means, and/or standard deviations for reporting the distribution of variables and the demographic factors, as well as to present findings for RQ1a–d and RQ2. Lastly, the ANCOVA technique was applied to produce findings for RQ1a–d and RQ2, with gender and weight status as fixed factors in the equations.

## 4. Results

Exploratory factor analysis results showed that 83.12% of the variance was explained in the equation by the items that measure “perceived ease of use” (Cronbach’s alpha = 0.90). Next, the factor analysis indicated that the measurement items for the “perceived usefulness” accounted for 81.86% of the variance (Cronbach’s alpha = 0.90). The third factor analysis equation had 86.91% of its variance explained by the items that assess “perceived usability” (Cronbach’s alpha = 0.93). Lastly, the items gauging the “perceived user satisfaction” construct explained 92.91% of its variance (Cronbach’s alpha = 0.96). The same factor analysis procedure was applied to validate the health promotion message construct. Results showed that the equation accounted for 78.61% of the variance of the construct (Cronbach’s alpha = 0.79).

The frequency analysis generated descriptive statistics for RQ1a–d, which explored user evaluation of the four usability factors, including (a) ease of use, (b) usefulness, (c) learnability, and (d) user satisfaction, respectively. Computed on a 7-point facial hedonic scale, the mean values for ease of use (M = 6.32, SD = 1.22), usefulness (M = 5.77, SD = 1.42), learnability (M = 5.79, SD = 1.57), and user satisfaction (M = 5.64, SD = 1.86) were above 6.33 and appeared to be relatively high. Similarly, RQ2, which involved the perceived effectiveness level of the health promotion messages, had a mean value of 6.32 (SD = 1.21), suggesting a relatively positive informational learning experience through receiving the health promotion messages. See Table 1 below for the statistics reported above. 

The ANCOVA procedure provided the answers for RQ3a–d, which queried whether the perceived level of (a) ease of use, (b) usefulness, (c) learnability, and (d) user satisfaction is separately and positively related to perceived health promotion message effectiveness. Both gender and weight status were entered as fixed factors in the equation, with each perceived app usability factor as the covariate and perceived health promotion message effectiveness as the dependent variable. Results indicated that neither gender nor weight status had a significant main effect. By contrast, ANCOVA results showed that the four usability factors—ease of use (F(1,17) = 38.99, *p* < 0.001, partial η^2^ = 0.74), usefulness (F(1,17) = 35.52, *p* < 0.001, partial η^2^ = 0.72), learnability (F(1,17) = 50.12, *p* < 0.001, partial η^2^ = 0.78), and user satisfaction (F(1,17) = 52.22, *p* < 0.001, partial η^2^ = 0.79)—were all significant predictors of perceived health message effectiveness. See Table 2 below for all ANCOVA results reported above.

## 5. Discussion

The current study tested the usability of a novel mobile phone app prototype whose conceptualization was guided by the principles of behavioral change theories (BCTs) and evidence-based pediatric obesity prevention. These BCTs include the SDT, SCT, and priming theory. Our mobile app is both unique and novel, owing to the infusion of BCT principles in the content features, the inclusion of evidence-based motivational messages, the targeting of early adolescents, and the engineering of a developmentally appropriate information architecture. It is worth noting that these different considerations associated with the app design itself likely have played a role in the success of the app prototype, as evaluated by the adolescent study participants.

The app prototype was usability-tested in a middle school whose student population included a high percentage of low-income families with greater risk of unhealthy lifestyle and obesity. The usability testing results confirmed that the app was equally well received by study participants of different genders and self-reported weight statuses (normal vs. overweight/obese). Equally important, and perhaps even a more interesting finding from this study, is the overwhelming positive feedback for the embedded motivational messages that promote healthy eating and active living, consistent with our previous findings [10].

Specifically, the participants found the app prototype to be easy to use, useful, highly learnable, and enjoyable to use. A closer look at the four different dimensions of the usability concept reveals that more than 75% of the participants gave a strongly favorable evaluation in response to all usability measurement items. Of particular importance is the participant response to the questions about usefulness, which ask participants to report and monitor their daily habits/activities. Each of these measurement items was presented with relevant images associated with daily habits and activities that have an impact on their dietary health and physical fitness; these items were engineered to prompt participants to think about and monitor their health-related behaviors.

The participant responses to the perceived usefulness of the app’s content features reported here also suggest that our app prototype was a good platform for reporting and monitoring health-related behaviors. These results then support the need for providing affordances in app features—to enable early adolescents to become self-aware of those behaviors that will impact their health and to allow them to develop self-confidence and exert self-control over those behaviors. They also offer broad or proxy confirmation for the applicability of SDT and SCT that helped guide the app development.

Applying an innovative strategy to incorporate tailored feedback into the app prototype, different motivational messages that promote health and nutrition were automatically triggered in response to the participants’ self-reported behaviors (see Appendix A). These pop-up messages were evaluated by the study participants. The evaluation results reveal that an overwhelming majority (89–96%) of these participants gave the following responses: (1) they enjoyed receiving the messages, (2) the messages taught them something new about food and activity, and (3) the information received will help enable them to make healthier choices in the future. Furthermore, as these pop-up messages appear as informational cues, they also serve as a vehicle for cognitive priming embedded in this unique mHealth program, which is consistent with our internet-based tailored message program [10].

Using machine learning algorithms, an app like ours could continuously program different and more effective motivational messages to prime users at mealtime, grocery stores, playgrounds, gymnasiums, and other occasions that involve making an eating or exercising decision. Hence, repeated use of this type of app could offer an early adolescent user an opportunity to both learn and develop a healthy lifestyle over time. These findings thus present a broad confirmation for the empirical implications of the priming theory, which serves as the guidepost for the development of those motivational messages.

The current app prototype was developed with the hope that it could be utilized as a complementary tool to aid school-based nutrition and physical education. Previous research suggests that health apps are more effective when they are used within another setting, such as a school environment [32]. This was also why our project collaborated with schoolteachers by having registered dietitians visit the classroom—before and after the usability study was conducted—to demonstrate the complementary nature of our mobile app tool.

Overall, this prototype has provided us with an excellent platform for the next phase of the project, which aims to further improve the app features to deliver nutrition education that contains more interactive features to facilitate behavior change in our target audience. One approach to engage children would be to incorporate animated features that could demonstrate the nutritional value of a food, drink, or snack they selected in a fun way. For example, an animated sequence will show that the selection of a sugary drink is associated with the potential of a tooth decay for a child, while the selection of milk is accompanied by healthier teeth and bones instead.

### Limitations

Several study limitations are worth noting. First, this app prototype was built on the Android platform. In our next iteration of app development, the iOS platform will also be programmed for future usability testing. Second, the study participation rate depended on whether the students shared the informed-consent-related handouts with their parents/guardians. In the next phase, we will adopt the practice that places the researchers at after-school events to discuss the study with both parents and children, which could more effectively increase parent awareness of the project in advance and further the potential study participation rate [57]. Third, the current prototype limits the amount of freestyle text entry. An improved version of the current app should consider adding more freestyle text interface to enable users to freely express their thoughts and feelings. Lastly, response bias is a possibility regarding self-reported height and weight to estimate weight status, particularly if a child felt as if he or she needed to respond in a certain way. In the future, additional steps can be taken to avoid response bias by altering the format of measurement items and asking the same question in different ways.

## 6. Conclusions

Currently, there remains a gap between scientific literature and the mHealth programs available in the market [24,29,32,58,59,60,61]. Research suggests that relying on behavior change theories (BCTs) to guide the development of future mHealth programs will likely produce more effective mHealth programs for children and adolescents [29]. As the development of the current app prototype continues, behavior change techniques—such as goal setting, motivational messaging, outcome feedback and reinforcements, cues to action, behavioral contracting, and rewards—could be messaging and interactive features that incorporate the constructs of BCTs.

The current mobile app design implemented the recommendations advanced in the empirical literature by (1) incorporating BCT-guided strategies and behavioral targets [62], (2) building a developmentally age-appropriate app [63], and (3) forming an interdisciplinary team to develop the app [24,25]. In particular, our interdisciplinary team included researchers from health communication, health sciences, and computer science/engineering, who conceptualized an app design to meet social scientific rigor and sound information architecture principles. Future versions of the app prototype could also be evaluated using the Mobile App Rating Scale (MARS), as this tool helps rate the engagement, functionality, aesthetics, appeal, and quality of information provided in the program design to determine the quality of health apps [64].

## Figures and Tables

**Table 1:** Descriptive statistics for all variables.

	M	Md	SD	N
Usability Fadctors				
Perceived ease of use	6.32	6.67	1.22	27
Perceived usefulness	5.77	6.33	1.42	27
Perceived learnability	5.79	6.33	1.57	27
Perceived user satisfaction	5.64	6.67	1.86	27
Health Message Effectiveness	6.32	6.67	1.21	27

**Table 2:** Two-way ANCOVA results for perceived health message effectiveness.

	*df*	*F*	*p*	η^2^
Gender	1,17	0.08	n.s.	0.01
Weight Status	1,17	0.17	n.s.	0.01
Ease of Use	1,17	38.99	<0.001	0.74
Gender	1,17	0.22	n.s.	0.02
Weight Status	1,17	0.62	n.s.	0.04
Usefulness	1,17	35.52	<0.001	0.72
Gender	1,17	1.83	n.s.	0.12
Weight Status	1,77	0.26	n.s.	0.02
Ease of Use	1,17	50.12	<0.001	0.78
Gender	1,17	1.15	.n.s.	0.08
Weight Status	1,17	0.08	n.s.	0.01
Ease of Use	1,17	52.22	<0.001	0.79

Note: Gender = Male vs. Female; Weight status = Normal (5 to <85 percentile) vs. Overweight/obese (≥85 percentile).

## Data Availability

The data presented in this study are not available as they were not part of the internal review board approval.

## Appendix A. Nutrition Messages for the EAMAIL App

**Directions:** A message will pop up when the user presses the “next” button. The message will be tailored to the item the user selected. If the user selected more than 1 item, only the item ranked highest on the list will appear. The items are ranked with 1 being the highest priority.

- Activity favorite → *since this is their favorite activity, we want the message to pop up for their ***first*** choice (this is ***not*** a ranked list)
  1. Outdoors—Some fresh air!
  2. Sports—Keep Movin’   
  3. Swimming—Like a Fish!
  4. Bike—Wheeling!
  5. Reading—Flipping pages!
  6. Cell phone—Connecting!
  7. TV—Zoned out!
  8. Music—Keep dancing!  
  9. Video games—Reality Check!
- After school (sedentary activities)→ All users will receive this same message:
- Try to be active and limit screen time to less than 2 h a day
- Keep studying & keep movin’
- Beverage favorite → *since this is their favorite activity, we want the message to pop up for their ***first*** choice (this is ***not*** a ranked list)
  1. Milk
    ■ Got milk? Go Bones!!
  2. Soda
    ■ Too sweet, watch your teeth
  3. Energy drinks
    ■ Too much buzz
  4. Sports drinks
    ■ Refuel with water
  5. Juice
    ■ Eat fruit—Yum!
  6. Water
    ■ Great Drink!!!
  7. Hi-C
    Try juice!
  8. Coffee
    Just one’s plenty
- Body image—what they think they look like
  1. **NO MESSAGE**
- Fast food → *Everyone who selects one of the pictures will receive the same message:
  1. Try eating at home too!
- Food favorite → *since this is their favorite activity, we want the message to pop up for their ***first*** choice (this is ***not*** a ranked list)
  1. Vegetables
    ■ Veggies Rule!
  2. Fruit
    ■ Eat the Rainbow!
  3. Bread
    ■ Go for Whole Grains!
  4. Cookies  
    ■ Not the cookie monster!
  5. Chicken
    ■ Build strong muscles!
  6. Fries
    ■ Try baked or mashed.
  7. Yogurt
    ■ Build strong bones!
  8. Cheese
    ■ Go calcium!
  9. Take out—Chinese food
    ■ Try eating at home too!
- Do you eat when you wake up?
  1. Yes
    ■ Nice work!
  2. Drink
    ■ Stay hydrated!
  3. No
    ■ Energize with breakfast!  
- What you eat first—Breakfast
  1. Fruit
    ■ Eat the Rainbow!
  2. Eggs
    ■ Incredible edible egg!  
  3. Yogurt
    ■ Yay calcium!  
  4. Breakfast sandwich
    ■ Go for Whole Grains!
  5. Bars
    ■ Add some fruit!   
  6. Cereal
    ■ Top it off with a !
  7. Baked goods
    ■ Go for Whole Grains instead!
  8. Chips
    ■ How about some fruit?
  9. Lunch food
    ■ Eat a balanced meal!  
- Breakfast drink
  1. Water
    ■ Great choice!  
  2. Milk
    ■ Got milk? Go Bones!!  
  3. Orange juice
    ■ Get your vitamin C!
  4. Chocolate milk
    ■ Got milk? Strong bones!!  
  5. Juice
    ■ How about water?
  6. Fruit punch
    ■ Try juice!
  7. Sports drink
    ■ Try water!  
  8. Soda
    ■ Too sweet, watch your teeth
  9. Nothing
    ■ Remember—stay hydrated!  
- Dessert → ** Everyone who selects one of the pictures will receive the same message:
  1. Try fruit for dessert!  
- Dinner
  1. Veggies
    ■ Awesome!  Go veggies!
  2. Chicken
    ■ Build strong muscles!
  3. Steak
    ■ Build strong muscles!
  4. Sandwich
    ■ Solid choice!
  5. Soup
    ■ Build a balanced plate
  6. Rice and beans
    ■ Great combo!  
  7. Italian
    ■ Go for Whole Grains!
  8. Fast food
    ■ Try eating at home too!
  9. Breakfast
    ■ Add some fruits and veggies!
- Dinner beverage
  1. Water
    ■ Great for your body!
  2. Milk
    ■ Got milk? Strong bones!
  3. Orange juice
    ■ Get your vitamin C!
  4. Chocolate milk
    ■ Got milk? Strong bones!
  5. Juice
    ■ Try whole fruit!
  6. Fruit punch
    ■ Try 100% fruit juice!
  7. Sports drink
    ■ Refuel with water!
  8. Soda
    ■ Too sweet  Try water!
  9. Nothing
    ■ Remember—stay hydrated!  
- Lunch
  1. Veggies
    ■ Veggies are awesome!  
  2. Fruit
    ■ Fruit’s the best!
  3. Yogurt
    Nice!  Strong bones!
  4. Sandwich
    ■ Yum! All the food groups!
  5. Pizza
    ■ Yum!  Try adding veggies!
  6. Lunchable
    ■ Make a balanced plate!
  7. Granola bar
    ■ Great snack!
  8. Chips
    ■ Go for Whole Grains!
  9. Cookie
    ■  Cut back on sweets—try fruit Cut back on sweets—try fruit!
- Lunch drink
  1. Water
    ■  Great choice!
  2. Milk
    ■ Got milk? Strong bones!
  3. Orange juice
    ■ Power to fight colds!
  4. Chocolate milk
    ■ Strong bones!
  5. Juice
    ■ Try whole fruit!
  6. Fruit punch
    ■ Too sweet  Try water!
  7. Sports drink
    ■ Stay hydrated with water!
  8. Soda
    ■ Watch the sugar
  9. Nothing
    ■ Remember—stay hydrated!  
- Snack→ ** Everyone who selects one of the pictures will receive the same message:
  1. Fruits & veggies are the best snacks!  
- Snack drink
  1. Water
    ■ Excellent choice!
  2. Milk
    ■ Got milk? Strong bones
  3. Orange juice
    ■ Packed with vitamin C!  
  4. Chocolate milk
    ■ Strong bones!
  5. Juice
    ■ Try whole fruit!
  6. Fruit punch
    ■ Why not juice?
  7. Sports drink
    ■ Drink water!
  8. Soda
    ■ Too sweet, watch your teeth
  9. Nothing
    ■ Carry a water bottle!
- Feeling (in the “about me” survey)
  1. The 3 **happy** faces will be grouped together to receive the same message. The 3 **unhappy** faces will be grouped and receive the same message.
    ■ Happy (1 of the 3 happy faces)
      - Stay smiling!
    ■ Sad (1 of the 3 unhappy faces)
      - Tomorrow is a new day!
- Sleep → Everyone will receive the same message, no matter what number they put.
  1. Try for 8 h a night!
